# Transforming Cancer Nanotechnology Data Analysis and User Experience. Part II: Providing Future Solutions Using Large Language Models

**DOI:** 10.1002/wnan.70029

**Published:** 2025-08-21

**Authors:** Weina Ke, Rui He, Mark A. Jensen, Marina A. Dobrovolskaia

**Affiliations:** ^1^ Bioinformatics and Computational Science, Frederick National Laboratory for Cancer Research Sponsored by the National Cancer Institute Frederick Maryland USA; ^2^ Essential Software, Inc Potomac Maryland USA; ^3^ Nanotechnology Characterization Laboratory, Cancer Research Technology Program, Frederick National Laboratory for Cancer Research Sponsored by the National Cancer Institute Frederick Maryland USA

**Keywords:** caNanoLab, characterization, data mining, data repository, large language models, nanomedicine

## Abstract

The advances in cancer nanotechnologies and current efforts focused on data sharing, along with the associated challenges, have been summarized in the first part of this review. Herein, we explore the potential of Large Language Models (LLMs) to enhance user experience, using the federally funded data repository caNanoLab as a case study. By training the LLM on caNanoLab data, we demonstrate its ability to provide more comprehensive search results, well‐guided data entry, and a personalized, assisted search experience. We also discuss how integrating LLMs would optimize user experience, making it easier for researchers to navigate complex data and find relevant information. Our findings suggest that LLMs have significant potential to transform cancer nanotechnology data analysis and user experience, potentially paving the way for more efficient and effective advancements in cancer research.

This article is categorized under:

Nanotechnology Approaches to Biology > Nanoscale Systems in Biology
Therapeutic Approaches and Drug Discovery > Emerging Technologies

## Graphical abstract

The first part of this review discussed challenges related to data complexity, management, standardization, privacy, and awareness, and how data repositories, such as caNanoLab, help users access large datasets related to nanomedicine characterization. In this section of the review, we discuss the application of Large Language Models to enhance user experience when mining large datasets in public data repositories.

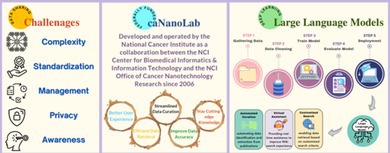

## 1 Introduction

A large language model (LLM) is a sophisticated deep‐learning algorithm that has been expertly designed to comprehend, process, and generate human language. LLMs are capable of comprehending, processing, and generating human language, with the ability to understand context and produce coherent responses that align logically with human thought processes. The underlying mechanism that fuels these language models involves analyzing colossal volumes of textual data, including books, articles, and websites, which are then used to identify and learn statistical patterns of language usage. These patterns are then employed to craft prose that is virtually indistinguishable from something that could have been written or spoken by a human being (Ibrahim 2024). LLMs also utilize advanced statistical techniques to recognize patterns and connections within language, enabling them to effectively capture the nuances and complexities of human communication (Abdo 2022). From text generation and machine translation to sentiment analysis and question‐answering, LLMs are highly versatile and have revolutionized the field of natural language processing (NLP). Their remarkable power and potential have recently propelled them into the spotlight as an invaluable tool for NLP research, marking a significant leap forward in the development of cutting‐edge language technologies.

One of the most notable models in recent years was the GPT (Generative Pre‐trained Transformer) model, created by OpenAI in 2018 (Mao 2023). The GPT model stood out because it was one of the first language models to use the transformer architecture (Ibrahim 2024). This type of neural network excels at identifying connections between words and phrases, even when they are far apart in a text. As a result, the model was able to generate language output in response to arbitrary prompts that was both coherent and contextually appropriate. Parameters are critical settings within an artificial intelligence (AI) model that can be extracted from input data to determine its predictive capabilities. In fact, the number of parameters an AI model has is a widely used measure of its performance. This measure stands out in groundbreaking models like GPT‐1, which boasted an unprecedented 117 million parameters (Babich 2022). Since then, more advanced language models have been developed, including GPT‐2, GPT‐3, and BERT. These models can generate complex and human‐like text, surpassing the capabilities of the original GPT model. ChatGPT was developed using GPT‐3, which has 175 billion parameters (Babich 2022). More recently, OpenAI has unveiled its latest model in the GPT series: GPT‐4.

This model has 100 trillion parameters (Babich 2022). Like its predecessor, GPT‐'s strength lies in its pre‐training on an enormous corpus of text data, which has allowed it to learn a diverse range of language features and relationships. However, GPT‐4 is 500 times more potent than GPT‐3, which was a powerful language model used to create ChatGPT (Ibrahim 2024). With this advance, we can expect more precise responses from GPT‐4, which may have an impact on the way we interact with artificial intelligence, potentially leading to even more personalized and accurate experiences.

Use of LLMs has led to substantial breakthroughs in natural language processing tasks. By relying on mathematical principles such as probability and linear algebra, LLM is able to recognize patterns in large datasets, generate predictions about new data, such as the probability distribution of words in a sentence, and generate new sentences based on this distribution (Princeton University cos126, n.d.). Expanding on LLM's success in natural language interface design in the domain of computer‐assisted creativity, it has demonstrated its capacity to generate coherent and contextually relevant text, including poetry on a wide range of topics (Chakrabarty et al. 2022). LLM has played a transformative role in programming by automating the generation of high‐quality program code, introducing a novel “democratic” approach to programming. LLMs can assist with various aspects of programming, including planning and debugging, providing valuable support in the form of an intelligent compiler, pair programming partner, or search‐and‐reuse feature (Sarkar et al. 2022). In addition to these notable achievements, LLMs have demonstrated exceptional progress in machine translation tasks, particularly for low‐resource languages (Moslem et al. 2023). LLMs have also proven invaluable in accurately classifying sentiment, thereby facilitating practical applications, such as social media monitoring (Kaliyar et al. 2021) and customer feedback analysis (Wen et al. 2023). In addition, LLMs have enhanced database functionality by improving search capabilities through natural language query comprehension, resulting in more relevant results to the user (Zhou et al. 2022). They can facilitate question‐answering systems (Huang et al. 2022), which enable users to ask natural language questions and receive accurate answers by extracting relevant information from databases using LLMs.

**FIGURE 1:**
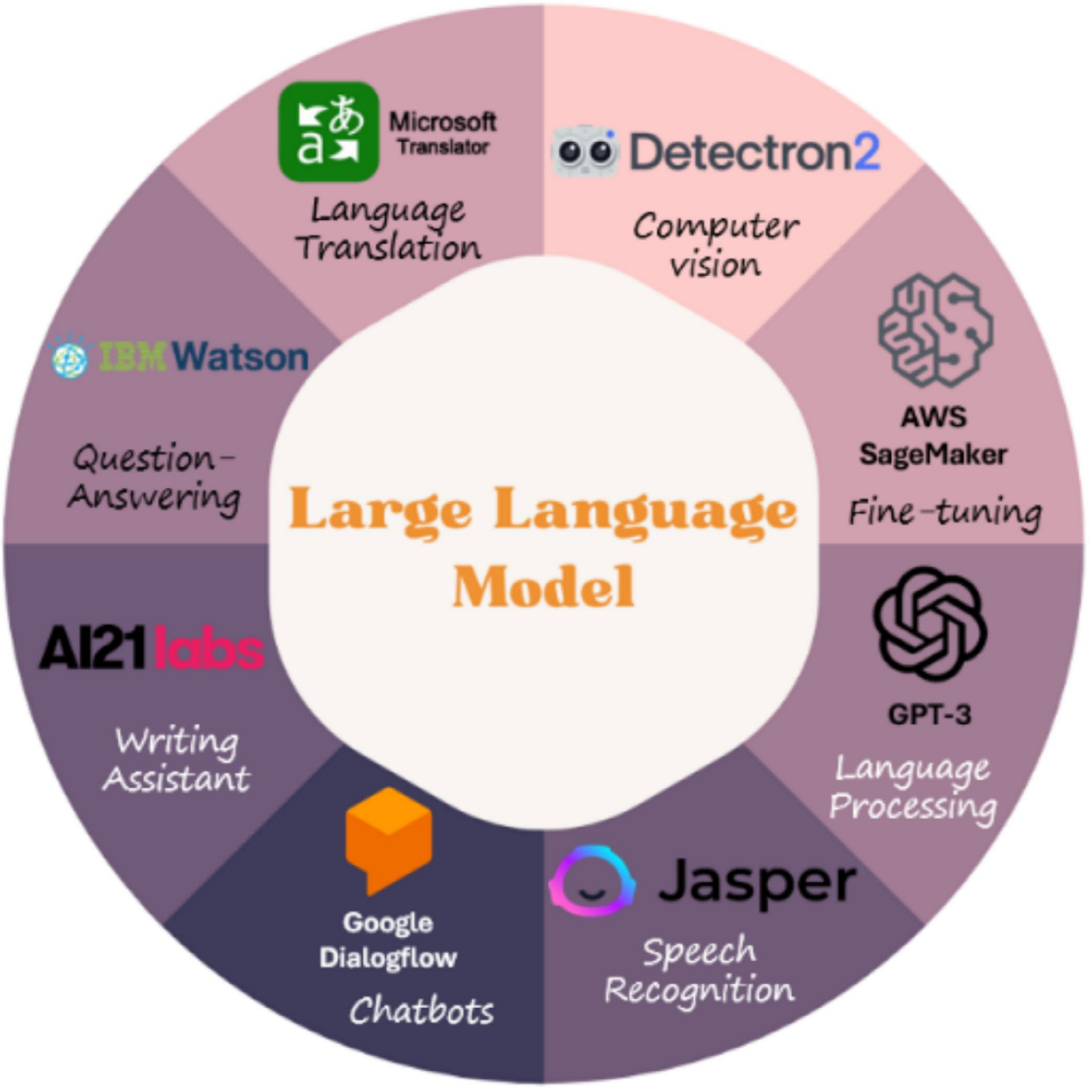
Examples of applications of the large language model. Examples of various applications of LLMs are summarized.

As LLMs gain popularity and power, they have achieved significant milestones across a wide range of industries. It is highly likely that many of us have interacted with an LLM without even realizing it, perhaps during a conversation with a virtual assistant or digital assistants (like Siri and Alexa), chatbots, language translation systems (like Google Translate), and others. In the medical field, LLMs are making their mark. For example, Caption Health is an FDA‐cleared product. Its AI‐guided ultrasound technology recognizes patterns in medical images using LLM, providing real‐time guidance to healthcare professionals during ultrasound examinations (NS Medical Staff Writer 2020). Another FDA‐cleared product, Aidoc, utilizes LLM to help radiologists identify and prioritize critical cases from large volumes of medical imaging studies like CT scans, MRIs, and X‐rays (Olsen 2022). IBM Watson for Oncology is trained on a large corpus of medical literature, including textbooks, medical journals, and clinical studies, and is constantly updated with new information (Liu et al. 2018). It can analyze patient data, including medical records, test results, and pathology reports, and provide treatment options tailored to the patient's specific diagnosis, medical history, and individual circumstances. The system has been deployed in hospitals and clinics worldwide and has been utilized to support oncologists in making informed treatment decisions for their patients. LLMs can be applied in various contexts, including language translation, speech recognition, and computer vision (Figure 1). These examples illustrate the versatility of LLMs and their potential to streamline and improve various tasks related to natural language processing and information retrieval.

## 2 The Potential of LLMs to Revolutionize Data Sharing and Management

With the increasing amount of data being generated in today's world, data sharing and management have become an urgent need for organizations across all sectors. Traditional data management methods, such as manual data entry and database querying, are often time‐consuming and prone to errors. There is a growing interest in developing new, more efficient methods for managing and sharing data. LLMs offer a potential solution by using natural language processing techniques to analyze and generate text data. Some ways that LLMs could potentially revolutionize data sharing and management are reviewed further below.

Natural language‐based data querying: By leveraging the natural language processing capabilities of LLMs, users can articulate queries in plain language, thereby surpassing the limitations of predefined search options and filters offered by search interfaces. This approach empowers users to ask questions, specify conditions, and provide contextual information in their own words, fostering enhanced flexibility and expressiveness in retrieving desired information (Bazaga et al. 2021). LLMs with natural language processing capabilities excel at comprehending and analyzing the intricacies of human language, enabling accurate interpretation of user queries. They translate natural language questions into formal database queries expressed in query languages such as SQL, which are then passed to the database query engine (Sun et al. 2023). By harnessing LLMs, websites can enhance their search capabilities, delivering a more intuitive and personalized data querying experience. Applications based on LLMs can offer a user‐friendly and streamlined data querying experience, accessible to users of all technical levels.

Data extraction, categorization, summarization, and validation: LLMs are particularly well‐suited for data management, as they leverage natural language processing techniques to extract and categorize data in a human‐like manner. By analyzing the text and identifying important semantic entities and relationships, LLMs can automatically extract and organize data from a variety of sources, including unstructured data such as emails and PDFs, saving valuable time and resources (Guevara et al. 2024). Furthermore, LLMs are capable of categorizing data based on similarities, such as topics or entities, which streamlines the management of large datasets and expedites access to relevant information (Wang et al. 2023). Additionally, LLMs summarize large volumes of data into shorter, more manageable, and computable formats (Tang et al. 2023). By producing concise and informative summaries, LLMs help users to prioritize relevant data and optimize decision‐making. Finally, LLMs can analyze data for errors or inconsistencies and correct them; for example, by identifying and correcting incorrect file names, file formats, or file locations as part of the data processing process. Furthermore, LLMs can identify any missing data, a crucial consideration for organizations grappling with vast datasets, as missing data can severely compromise the validity of research outcomes.

### 2.1 Data Augmentation

Data augmentation refers to the process of artificially increasing the size and diversity of a dataset by generating new data points from the existing data (Shah 2023). This is particularly advantageous when there is limited available data or concerns regarding the privacy of raw data. This approach enables users to expand their datasets in a controlled and secure manner while preserving the privacy of sensitive information (Yuan et al. 2023). Using synthetic data also allows LLMs to assist in detecting and mitigating biases in existing datasets, thereby improving the accuracy and fairness of the data. The synthetic data could be statistically similar to the original data, yet it is devoid of any personally identifiable information (PII). This feature is attractive for data analysis without compromising privacy.

### 2.2 Data Translation

Data translation refers to the process of converting data from one format or language to another. This is a common challenge in data management, particularly when dealing with large datasets that may be stored in non‐standard formats or include data in multiple human languages. Using LLMs to automate the translation process makes managing data across different formats and languages more straightforward and efficient. LLMs like GPT‐3 and others are not just adept at translating between widely spoken languages, but they also show promise in handling less common languages, a significant step in making data accessible across linguistic barriers (Zhu et al. 2023).

### 2.3 Natural Language Generation (NLG)

NLG refers to the process of generating natural language text or speech from a dataset. NLG is a powerful application of LLMs, as it enables the generation of natural language descriptions of complex data. This technology is a valuable tool for organizations, allowing them to communicate the findings of large datasets to non‐experts or create reports and summaries of data in an easily understandable format. LLMs are able to transform complex data into easily understandable and informative representations, enabling stakeholders to gain valuable insights and make informed decisions (Naveed et al. 2023).

LLMs are the basis of a versatile and advanced toolset for enhancing data management and data sharing practices. They provide various methods to improve data accessibility, comprehensibility, and utility for a broader user base, ultimately leading to increased efficiency and accuracy. In addition to the previously mentioned benefits, LLMs also enhance user experience and data exploration. By optimizing user interfaces and providing informative natural language chatbots, LLMs can improve the accessibility and usability of data, making it easier for researchers to access relevant information. Furthermore, LLMs can provide powerful data exploration tools that enable researchers to analyze complex data in real‐time, facilitating informed decision‐making. We will delve into these topics in greater detail.

## 3 LLMs for Enhancing User Experience

With the exponential growth of data across almost all fields, the task of navigating multiple databases with different user interfaces has become increasingly daunting. Current data extraction and organization methods often require significant manual effort and are prone to error. Additionally, many databases are poorly organized, featuring unintuitive interfaces or overly complex search mechanisms. These factors can lead to frustrated, overwhelmed, and discouraged users, who invested a substantial amount of time without retrieving the relevant data. Leveraging LLMs holds the potential to significantly enhance the user experience related to cancer nanotechnology and beyond. Firstly, LLMs analyze and process vast amounts of data quickly and accurately. Such efficient and effective search reduces the time and effort required by users to manually sift through large amounts of data, increasing their productivity and satisfaction. Secondly, by identifying and extracting key information from unstructured data, LLMs provide users with more relevant search results that match their research interests, thereby reducing the effort required to filter out irrelevant or incomplete data. Thirdly, LLMs help create more intuitive and user‐friendly interfaces, such as chatbots or voice assistants, that enable users to interact with the database more naturally and intuitively, thereby reducing user confusion and difficulty in navigating complex interfaces or search mechanisms.

Even if users are successful in retrieving the data they need, accessing all relevant information can still be a challenge. For instance, users may need to click on each search result to view additional details, such as the abstract or full text. This can be time‐consuming and frustrating, especially for those who want to quickly scan the search results to identify relevant articles. Additionally, not all databases come equipped with presentation tools. Among those that do, the tools may not be customizable, which can limit users who want to customize the view according to their specific research interests or preferences. Furthermore, some databases may provide visualization tools that are difficult to interpret or contain terminology and abbreviations that are hard to understand. This can further impede the user's ability to effectively utilize the database, leading to frustration and difficulty in obtaining relevant information. LLMs, on the other hand, can help overcome the above challenges by providing a more natural and intuitive user interface. LLMs can provide interactive visualizations and summaries of the data, making it easier for users to interpret and understand the information (Dibia 2023). LLMs also improve the accuracy and consistency of data presentation (Raj et al. 2023). With the ability to automatically identify and extract key information, LLMs ensure that the same information is presented consistently across different data sources, reducing the likelihood of errors or inconsistencies (Anonymous ACL submission 2023). Additionally, LLMs can be utilized to standardize and harmonize data, thereby facilitating easier comparison and integration of data from various sources (Sedinkina 2023). This can help improve the quality and reliability of the data being presented, thereby enhancing the overall user experience.

## 4 LLMs for Enhancing Data Exploration

LLMs are powerful tools that enable efficient exploration and analysis of large datasets in various fields (Panuganty, n.d.). These models are particularly useful for identifying trends and patterns in complex and unstructured data (Panuganty, n.d.). A key feature of LLMs is their ability to process natural language, which can be leveraged to improve data exploration in a wide range of research topics and business domains. This means these models can understand and analyze human language, allowing researchers to input large amounts of text data, such as scientific articles, and extract relevant information (QUOTIUM RESEARCH CENTER, n.d.). For example, within clinical trials, LLMs can be used to interpret extensive scientific texts and reports, enhancing patient‐trial matching, trial planning, and the extraction of relevant information, thus streamlining and improving the efficacy of clinical trial processes (Ghim and Ahn 2023).

Another way that LLMs enhance data exploration is to generate new hypotheses and predictions based on existing data (Powell 2023; Qi et al. 2023). These models are adept at discerning patterns and relationships in extensive datasets, enabling them to unveil new insights and identify areas ripe for further research. Their capabilities extend to diverse applications, such as proposing novel hypotheses in biomedical research or facilitating scientific discovery by generating hypotheses from interdisciplinary knowledge (Qi et al. 2023). Additionally, LLMs are utilized to identify knowledge gaps and suggest new research directions. For instance, in the context of clinical knowledge evaluation, LLMs are employed to uncover gaps in our understanding of medical information (Singhal et al. 2023).

Additionally, they guide future research directions by revealing areas where LLMs can be enhanced for more accurate and reliable clinical applications (Singhal et al. 2023). This can help researchers prioritize their efforts and focus on areas where they are most likely to make significant contributions. As these models continue to advance, they are expected to play an increasingly important role in accelerating research findings and providing valuable insights into various disciplines, thereby enabling informed decision‐making and staying ahead of the curve. Examples of improved real‐time data exploration, visualization, and analytics are demonstrated in the following examples.

Natural Language Processing: In the realm of oncology, the deployment of LLMs marks a revolutionary shift towards advanced, nuanced data analysis. These sophisticated models excel in sifting through and interpreting vast datasets found in electronic medical records, unearthing clinical information essential for accurate cancer diagnosis, effective staging, and precise treatment evaluation. Their application goes beyond traditional data processing, enabling a deeper, more comprehensive understanding of patient profiles and treatment responses. This capability is particularly significant in the context of personalized medicine, where tailoring treatments to individual patients' needs is paramount. LLMs empower oncologists to make more informed decisions, which may lead to improved patient outcomes and pioneer new frontiers in cancer research and therapy (Puts et al. 2024).

### 4.1 Intelligent Chatbots

LLMs are revolutionizing customer service in e‐commerce and e‐services. These sophisticated chatbots are capable of much more than basic query responses; they excel in providing personalized recommendations and performing a variety of tasks by analyzing text data from customer interactions. Their real‐time response capabilities significantly enhance customer engagement and service quality. For instance, an LLM‐driven chatbot can instantly address specific customer queries about products or services, offering a tailored and efficient customer experience. The integration of such chatbots represents a major step forward in meeting customer expectations and improving service performance, underscoring their vital role in modern customer service strategies (Misischia et al. 2022).

### 4.2 Predictive Analytics

LLMs are revolutionizing predictive analytics in the business sector. By analyzing complex data sets, LLMs can accurately forecast market trends and consumer behavior. Their proficiency in processing vast amounts of text data, such as sales figures and customer feedback, enables businesses to refine their strategies and decision‐making processes effectively. This technological advancement in predictive analytics can lead to a better understanding of market dynamics (Turing, n.d.).

### 4.3 Image and Video Analysis

The advancement of LLMs like ChatGPT has significantly enhanced medical image processing. These models have become essential in interpreting complex visual data, enabling more informed diagnostic and treatment decisions in healthcare. For instance, LLMs can efficiently process and analyze MRI or CT scans and reports, identifying subtle patterns indicative of disease that might be challenging for manual review. This capability is crucial for early and accurate diagnosis, especially in conditions like cancer, where early detection can significantly influence treatment outcomes (Tian et al. 2024).

### 4.4 Data Visualization

LLMs have been utilized to enhance complex data visualization, facilitating navigation of challenges such as understanding data semantics and establishing visualization goals. An exemplary tool in this realm includes modules for summarizing data into natural language, identifying visualization objectives, generating and refining visualization code, and creating stylized graphics using image generation models. For instance, it can turn a complex dataset into an engaging infographic, ensuring the visualization is both informative and visually appealing. This innovative approach not only democratizes data visualization, making it accessible to a wider audience, but also fosters a deeper engagement with data through visually compelling and insightful narratives (Bollegala et al. 2023).

## 5 Example Applications of LLMs in caNanoLab

### 5.1 Automated Curation Capabilities in caNanoLab Using State‐Of‐The‐Art Natural Language Processing

Integrating advanced NLP techniques, particularly those based on LLMs, holds the promise of significantly improving the precision and effectiveness of scientific data curation in caNanoLab. NLP techniques are especially advantageous for this task for two main reasons:

Firstly, LLMs have the capability to analyze vast amounts of unstructured text data and extract pertinent information with notable accuracy. Secondly, the intrinsic structure of scientific papers, which typically includes well‐defined content sections, provides an indexed format that facilitates information extraction. To realize this potential, the process of automated curation can be broken down into four essential steps.

#### 5.1.1 Parsing Scientific Articles

**FIGURE 2:**
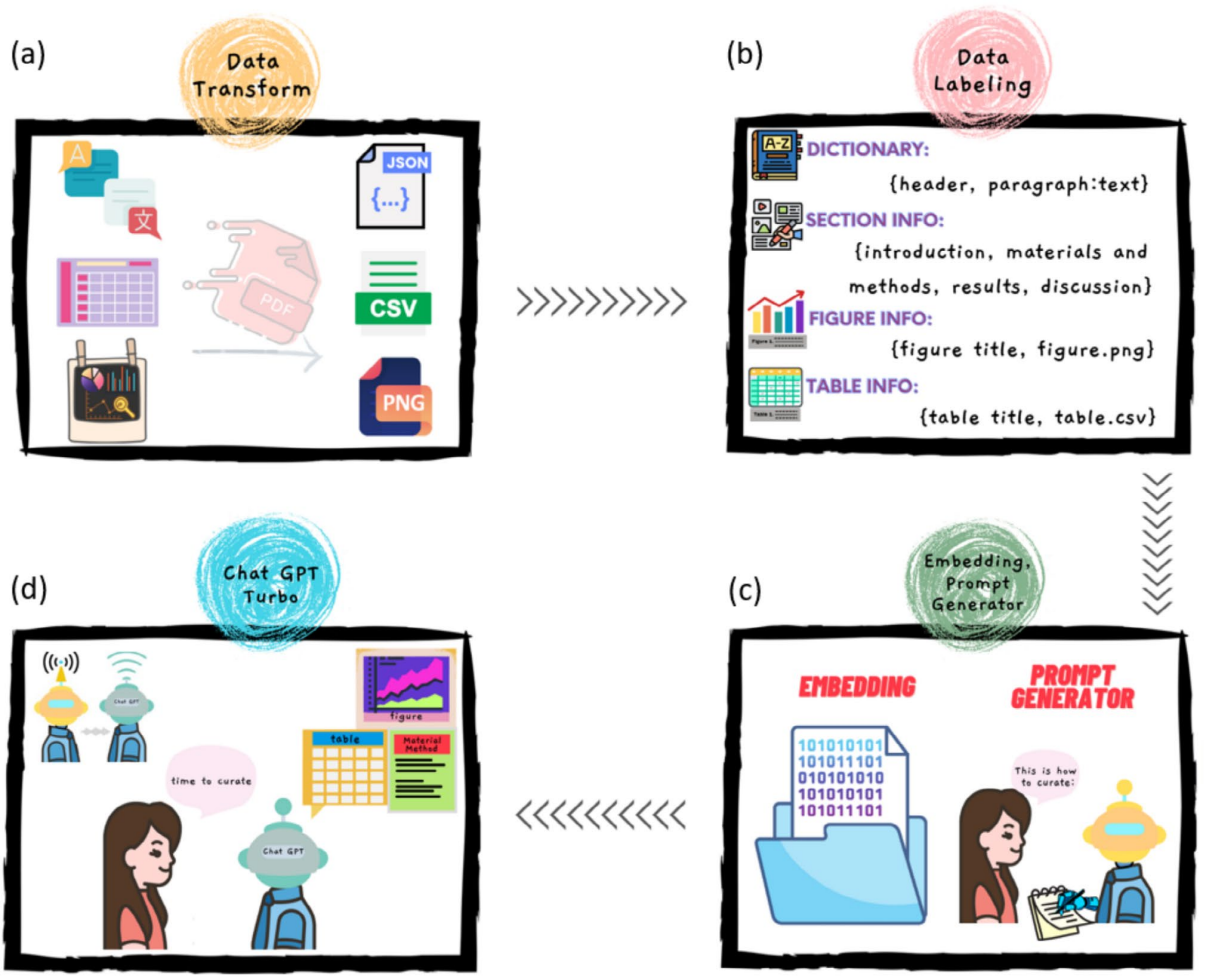
Four steps to achieve automated curation. Steps for automated curation are summarized: Data transformation (a), data labeling (b), Chat GPT Turbo (c), and embedding and prompt generator (d).

The initial step involves parsing the scientific article into tabular data, image files, and textual content. Given that the input file is most likely to be in PDF format, it is transformed into an accessible data structure to allow further processing (Figure 2a). This step may not be necessary for publishers, as they would have access to the original content; however, for a general non‐profit public database, this can be a potentially challenging step. Another advantage of maintaining an inventory of source PDFs is that it allows curators to track content sources for quality checks or corrections.

#### 5.1.2 Disassembling and Labeling Textual Contents

The second step is to disassemble and label the comprehensive textual contents, such as the body of scientific articles, into indexed content sections. These components can include informative text paragraphs, such as introductions, materials, and methods, tabular data tables, or graphic files. While the relationship between these textual information pieces is preserved by their location in the article index, marked by the section header and header levels, the figure and table titles provide a concise summary of their content and relevance to the main text body. These labels or indices convey the relationship between information components for downstream processes (Figure 2b).

#### 5.1.3 Converting Text to Embedding Format for Prompt Engineering

The next step is to convert the textual information into an embedding format, which is a numerical representation of the text, for content search and prompt engineering. The prompt is the question string submitted to an LLM to get a response. While the LLMs are excellent at understanding natural language questions, the quality of their answers heavily depends on the question itself and, more importantly, how the question is asked. The method of generating a prompt that properly asks the question and defines what the answer should contain is called prompt engineering. Compared to traditional task‐oriented models such as a text parser or a database query, which would have a fixed set of rules around the input and output (I/O) and expected behavior, the output and performance of a LLM model on a specific question would depend on how the question is asked and what guidelines/constraints are given in the prompt. A good prompt template for extracting specific information limited to a certain context is to provide the context with the question and add constraints for LLMs to answer the question strictly from the given context. For example, a prompt to limit the search range and explicitly request the output format would conclude with the phrase “strictly from the provided context and return the answer in JSON format.” To perform such a search within the context of a given paper, we can convert the article content into an embedding file and utilize the vector similarity method to gather relevant information as context for creating a prompt (Tibshirani 2022). The embedding file is a vectorized presentation of the natural language contents. The vector similarity method is particularly useful for answering more general questions that involve information scattered throughout the article (Figure 2c). An example of such questions would be extracting the methods and findings associated with a figure in the article. In general, the method would be located in the Methods or Materials and Instruments section, depending on the field, and the findings could be included in the Results and Discussion section. Although technology is advancing, allowing for longer prompt lengths that enable the entire textual content to be provided as context for each question asked, providing a well‐engineered prompt with only relevant context from the article can significantly reduce resource consumption and processing time.

#### 5.1.4 Submitting Engineered Prompts and Formatting Responses

The final step is to submit the engineered prompts and format the answers in the designated formats. A standardized output from this step can benefit downstream processing, such as quality checks, summary reports, and data storage (Figure 2d). With these considerations in mind, we developed a preliminary application based on the four steps outlined above. We use resources from the OpenAI Cookbook, a community‐driven resource for accomplishing tasks with the OpenAI API (Yuan et al. 2023).

For step one, we used the Adobe Services API, which is powered by AI models, to convert the PDF file into formatted textual contents in JSON format, tables in CSV format, and images in PNG format. The JSON file preserves the article structure by exporting text levels (header levels, body, etc.) and the image/table filename in the position where the article appears. A parser script was developed to perform the second step, data labeling. This labeled dataset is a PANDAS data frame containing each sentence in the text body indexed by the first two header levels, and the dataset is converted to the embedding file for further processing (McKinney 2010). The LLM used for applications in this work is the ChatGPT API, utilizing the GPT‐3.5 Turbo model, as it was the most powerful publicly available LLM model at the time of app development. The model possesses a good zero‐shot classification ability, which enables it to recognize and categorize new inputs it has not previously encountered by understanding the relationships between different categories and accurately predicting to which category the new input is likely to belong (Clavié et al. 2023). This ability makes it an effective tool for classification tasks, including categorizing data into predefined structures within a manuscript.

While the curation process for caNanoLab data entry focuses on the images in figures and extracts methods and conclusions associated with an image, the questions for step three are built around the title and legend of each figure. Since the goal is to extract original sentences in the text body that are related to the figure, the prompt would be led by the general task requirement to: “Extract original sentences about material, protocol, experiment result, and experiment conclusion of:” *followed by the title and legend if exists, then constrained by the following*: “strictly from context for each subplot labeled like a,b,c… or 1,2,3… or i, ii, iii… Output the information into: {“*Figure”: “subplot”: “protocol”: “result”: “conclusion*”:} with UTF‐8 format. Separate each subplot with two new lines.”

The prompt constrains ChatGPT to extract literal information from the provided context, adhering to a strict output format. The context is established through the vector similarity search of the figure title and legends. The response from GPT such as {*“Figure”: “4”, “subplot”: “A”, “protocol”: “Example Protocol”, “result”: “example result”, “conclusion”: “example conclusion”*} is formatted into CSV format for data uploads and HTML for human inspection by a parser script.

**FIGURE 3:**
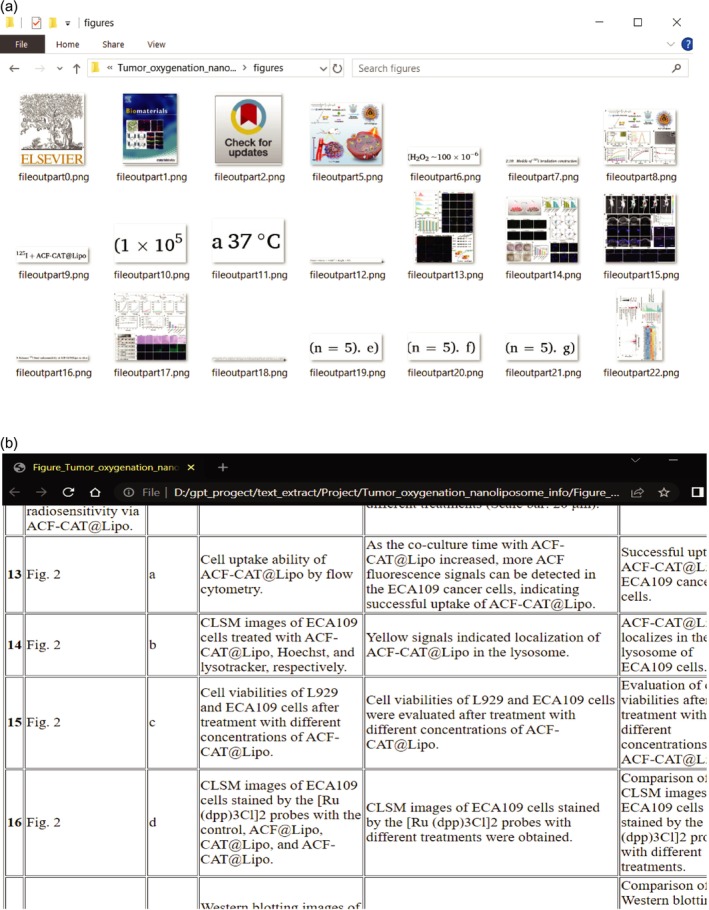
The demo of LLM generated figures. Figure title, legend, conclusion (a), and experiment method (b).

Step four was made easier by standardizing the response format of LLMs through prompt engineering, which generated several outcomes of the automated curation process (Figure 3). By leveraging LLM, we were able to generate desired outputs, including figures with titles, corresponding legends, conclusions, and experimental methods, thereby streamlining the curation process. Despite the advantages of the automated curation process, it is essential to acknowledge that this tool does not independently verify the scientific aspects of the input; this aspect of the work requires human input and expertise. By working in conjunction with human curators, this technology helps reduce manual labor and streamline processes, enabling curators to focus on verifying and evaluating the scientific aspects, ensuring accuracy and quality. This collaborative approach enables humans to focus on high‐demand tasks that require their critical thinking and domain‐specific knowledge. The computer‐aided process has certain limitations that can impede its accuracy. One such limitation is variability in the structure of research articles. Traditionally, articles follow a pre‐defined structure consisting of sections such as abstract, introduction, methodology, results, and conclusion. This standardized layout facilitates information retrieval and comprehension. However, if an article lacks a clear and structured format, extracting complete and accurate data becomes challenging. In cases where articles deviate from the expected organizational structure or contain ambiguities, the automated curation process may encounter difficulties. The absence of well‐defined sections or the presence of unclear delineations can hinder the extraction of information, making it more challenging to curate the data effectively. It becomes important to address these limitations by developing robust algorithms that can adapt to variations in article structure and effectively handle ambiguous cases. By recognizing these limitations and working towards refining the automated curation process, we can continue to enhance its accuracy and utility in facilitating data extraction and analysis.

### 5.2 Implement a Chatbot to Efficiently Assist Users With Specific Information and Guidance on caNanoLab Wiki

The caNanoLab Wiki is a web‐based platform established to support the effective use and development of caNanoLab. It is a part of the larger caNanoLab project, which is sponsored by the National Cancer Institute (NCI) and aims to advance cancer research by facilitating information sharing across the international biomedical nanotechnology community to expedite and validate the use of nanotechnology in biomedicine (NCI Hub, n.d.). The caNanoLab Wiki is a valuable resource for researchers and investigators seeking guidance on how to effectively utilize the caNanoLab website. However, due to the abundance of information available on the Wiki, users may sometimes struggle to find the specific information they need, which can negatively impact their overall user experience. An LLM‐enabled chatbot is an intelligent computer program that utilizes AI and NLP technologies to understand user input, simulate human conversation, and provide automated guidance and support on various online platforms (Caldarini et al. 2022).

Given the advantages of chatbots, such as rapid access to information, round‐the‐clock availability, personalized interactions, and streamlined processes, we implemented an initial chatbot using caNanoLab data as a tool for providing specific information and guidance on the caNanoLab Wiki with a more user‐friendly search experience by accepting natural language queries instead of technical jargon. Furthermore, the chatbot's ability to provide targeted and immediate responses can save users time and enhance their overall experience.

**FIGURE 4:**
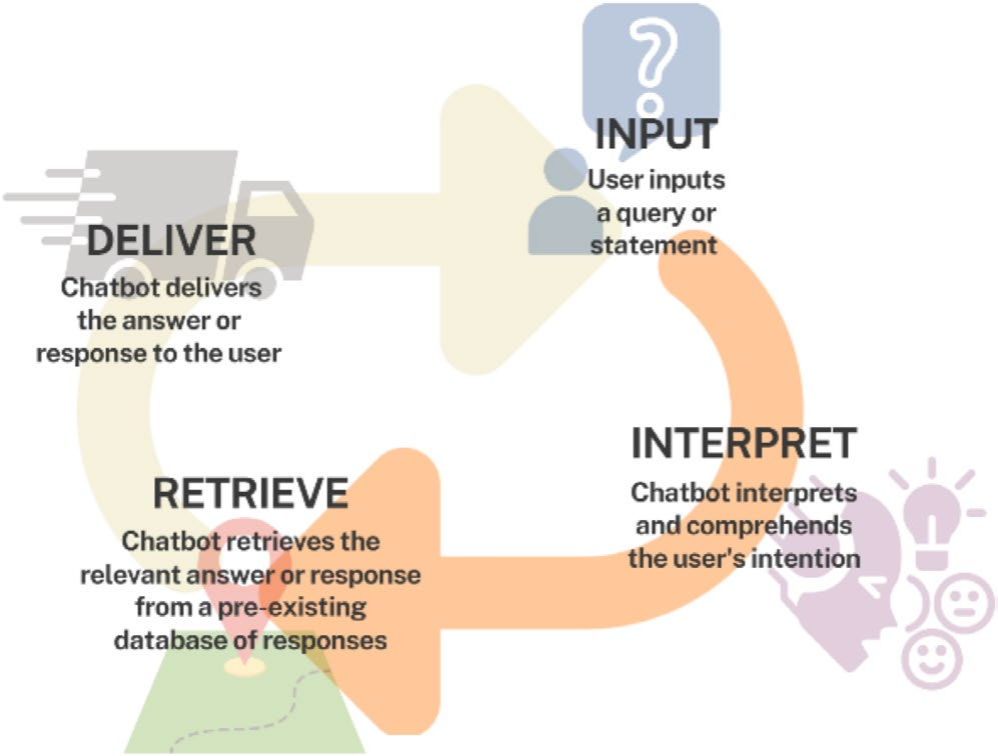
The workflow of the Chatbot. Essential steps of the workflow are summarized.

The design principle of the current chatbot on the specific knowledge base can be summarized in one sentence: retrieve only the information the user has asked about, directly from the designated knowledge pool to the user. Compared to typical content search, which requires precise wording and presents lengthy responses containing non‐relevant information alongside the desired information, the LLM enables users to ask questions in natural sentences and returns precise answers. The question‐and‐answer (Q&A) process will then become straightforward: the user inputs a question; the chatbot interprets and understands the input, retrieves the relevant information from a knowledge base to answer the question, and delivers the answer back to the user (Figure 4).

The general process for this LLM‐powered chatbot employs similar engineering concepts to those used in automated curation tasks, but now allows users to create questions (i.e., prompts) interactively. In the chatbot application, the knowledge base is limited to existing documentation, such as user manuals, existing wiki Q&A, and forum exchanges. This information can come in various forms and should eventually be converted to embedding files for context search and extraction. The prompt generator should take in the question from the user, search and add relevant context to the prompt, and limit the answer to the source only from the provided context to avoid hallucination (Zhang et al. 2023). The LLM model in this application serves as an assistant who presents the context in natural language to facilitate understanding. The answer is processed by a formatter to be presented to users in a readable manner on the interface.

**FIGURE 5:**
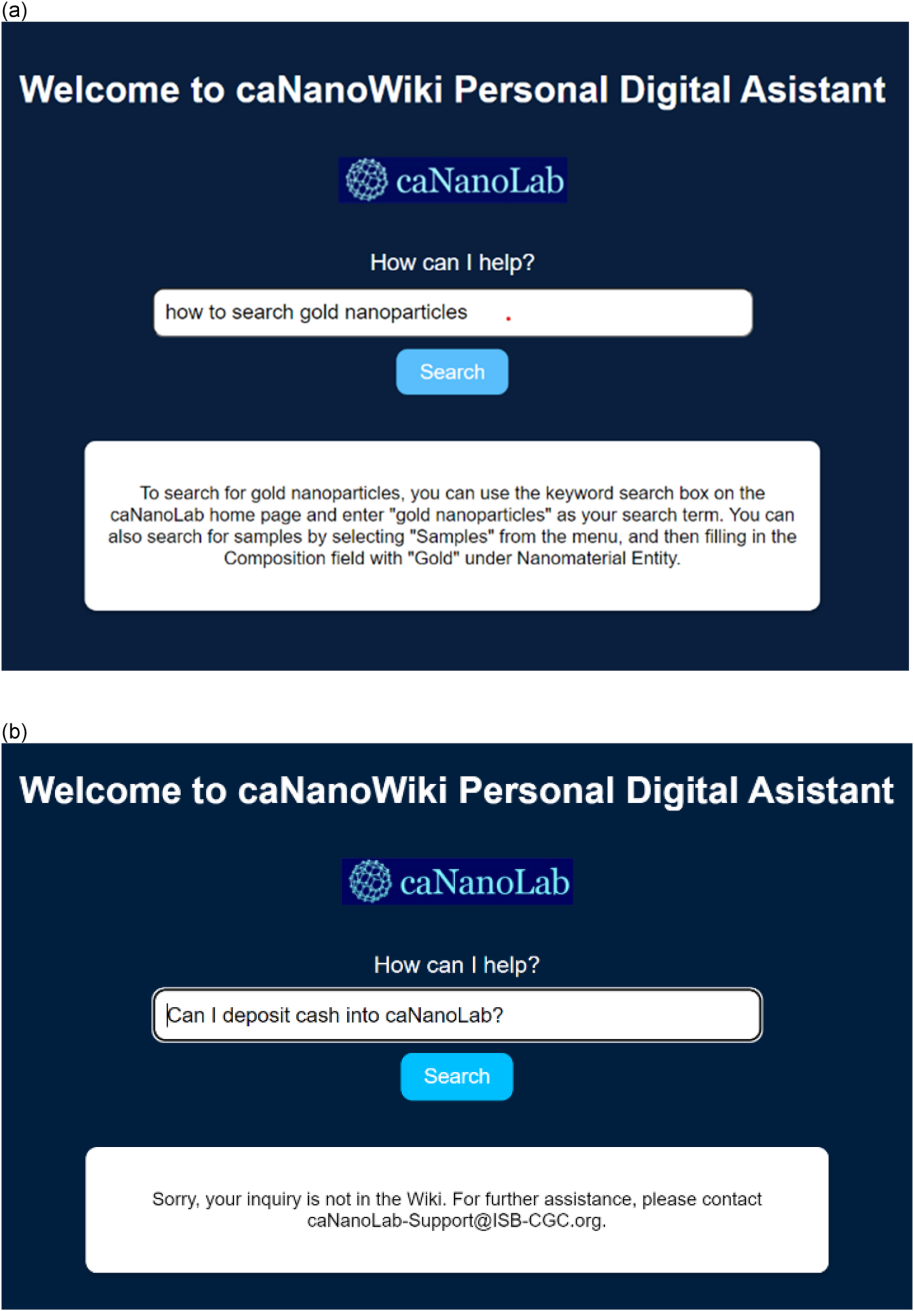
Personalized inquiry of caNanoLab Wiki. Example of caNanoLab Wiki output.

Herein, we present a demonstration of a personalized inquiry using caNanoLab Wiki (Figure 5). In the top panel, a user expresses their interest in accessing information specifically related to gold nanoparticles. The chatbot responds with detailed instructions, guiding the user on how to find the desired information within the Wiki. In the middle panel, we address user inquiries that extend beyond the scope of the Wiki. In such cases, the chatbot provides the contact information of our support team, who are readily available to provide further assistance. More importantly, in cases where users inquire about information that is not available within the Wiki, the chatbot will notify them that the requested information is unavailable and offer their contact information for further assistance, as demonstrated in the bottom panel. This ensures that users are aware of the limitations of the Wiki and can easily contact our support team for further assistance. This approach ensures that users can obtain information both from the extensive resources within the caNanoLab Wiki and our knowledgeable support team when needed.

The accuracy of the chatbot's responses relies on the context available within the Wiki. It is essential to recognize that if the desired information is not available in the Wiki, the chatbot will be unable to provide accurate answers. The accuracy and relevance of the chatbot's responses are also influenced by the timeliness and comprehensiveness of the Wiki's content. Hence, regular updates to the Wiki and the corresponding embedding file are essential to ensure the information remains current and reliable. To understand the functionality of caNanoWikiPDA and how it can aid in navigating caNanoLab Wiki, we invite you to explore the tutorial available in Section 5.4 at our beta site.

### 5.3 Deploy a Search Bar for Querying the caNanoLab Database via Natural Language

caNanoLab serves as a centralized repository housing an extensive collection of valuable data pertaining to cancer nanotechnology research. Currently, the repository indexes thousands of publications, samples, and hundreds of protocols, with these numbers continuing to grow as more data is added. The significance and impact of this dataset can only be fully realized if researchers and investigators can access and utilize it effectively. One key feature that facilitates data accessibility is the search function provided on the caNanoLab portal. However, the current search function has its limitations, offering only a limited set of search criteria. Given that researchers have varied interests and needs, a typical search function may not cater to everyone's needs. For instance, researchers interested in accessing publications from a specific time frame may encounter difficulties in finding the relevant information they need.

Therefore, caNanoLab requires a search mechanism that caters to the individual interests and needs of users, providing maximum benefit to the scientific community. An LLM‐based approach to search can offer a novel and user‐friendly way for researchers and investigators to retrieve information efficiently. Unlike the traditional method of selecting pre‐defined search criteria and navigating through the results, this new method enables users to retrieve data via natural language, making the search process more intuitive and streamlined. This search bar is expected to significantly enhance data accessibility and user experience, enabling researchers to find relevant information more effectively.

The conceptual design synopsis of this application aims to establish the infrastructure for delivering queries to the database, utilizing the LLM to generate formal search queries from natural language prompts and perform the search on behalf of the user. This core functionality requires the LLMs to know how to perform queries using the database language and have the necessary infrastructure to execute searches with the returned query string through function calls. This design will likely perform best on a database that can be queried with SQL, as this language has a large existing knowledge base, which is part of the open internet training material for current LLMs. If a relatively new database language is preferred for the targeting project, existing generic LLMs might not be able to generate an ideal query and, hence, may require model fine‐tuning. Fine‐tuning would require additional resources and sufficient training material and can potentially be a lengthy and expensive process. Along with the core query module, we need to create upstream guidance for the LLM, including documentation of the dataset structure, preparation instructions, and relevant notes for using the database. Additional components include the internal prompt generator and downstream infrastructure to render the dynamic data structure of query results to users. While database structures can be provided in standard formats to the LLMs, unstructured notes are used to provide specific guidance, such as clarifications of scientific terminology, additional information on tables and keys, or synonyms for certain terms. These notes, provided as context for the prompt, help the LLM understand the user's question. Updating the notes over time can be a potential way to enable the application to adapt to rapidly evolving research fields, potentially without requiring retraining of the models.

**FIGURE 6:**
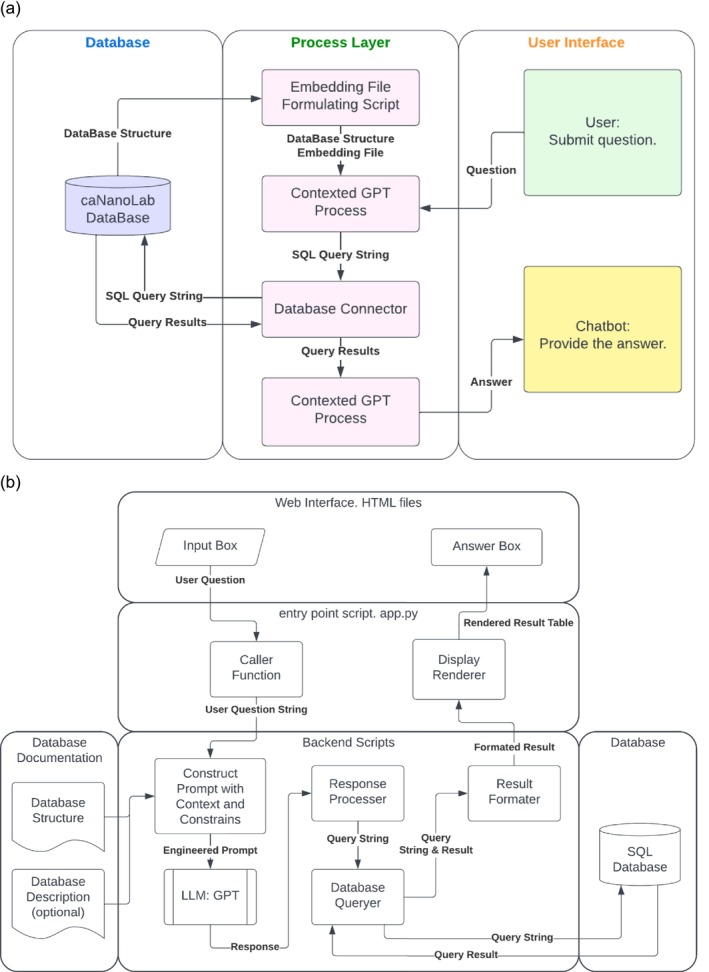
Workflow of the contextual query processing and database interaction in caNanoLab. Summarized in these diagrams are the relationships and interconnection between the database, process layer, and user interface (a), and a schematic of the workflow (b).

With these points in mind, we built the application caNanoLibrarian (Figure 6). The design employs three layers. The first layer is the graphical user interface (GUI), which takes questions from users and provides feedback to them. This layer defines how users interact with the application and what additional features or resources are available to them. Within the current scope of the work, this application comprises the following essential components: an input box for collecting questions, an output box for displaying results, a display region for showing the LLM‐generated query string, and a database structure section providing information to the user. The GUI uses the application layer to interact with the core function module and parse the responses for user presentation. This layer serves as the hub for all other potential features, such as data visualization tools, feedback collectors, or a search tool against a pool of previously submitted questions and their corresponding queries.

The third, backend layer, carries out the tasks mentioned above. Once the question is received by the prompt generator, the prompt generator adds the header: “select the appropriate table(s), write me a SQL query to:” connected by the question, and then the constraint: “return SQL query only.”, followed by the database structure and notes. Adding the entire database structure string as the context to each prompt adds a trivial cost to the LLM query. However, for a more complex database with significant web traffic, a potential way to reduce cost can be to vectorize questions and add related notes and database structures acquired from the vector similarity method, as was done in the Wiki application. The query is then passed to the database querying function to perform the search, and the search results are passed to the formatter to be returned to the application layer for rendering. This modular approach enables the application to expand without requiring a complete rebuild of the entire structure and infrastructure, as it was designed independently of the LLM. This allows any model of interest to be incorporated. It should be noted that each LLM may have different preferred words to acquire the best‐performing prompts; therefore, the prompt generator or prompt engineering methods should vary depending on the LLMs used.

**FIGURE 7:**
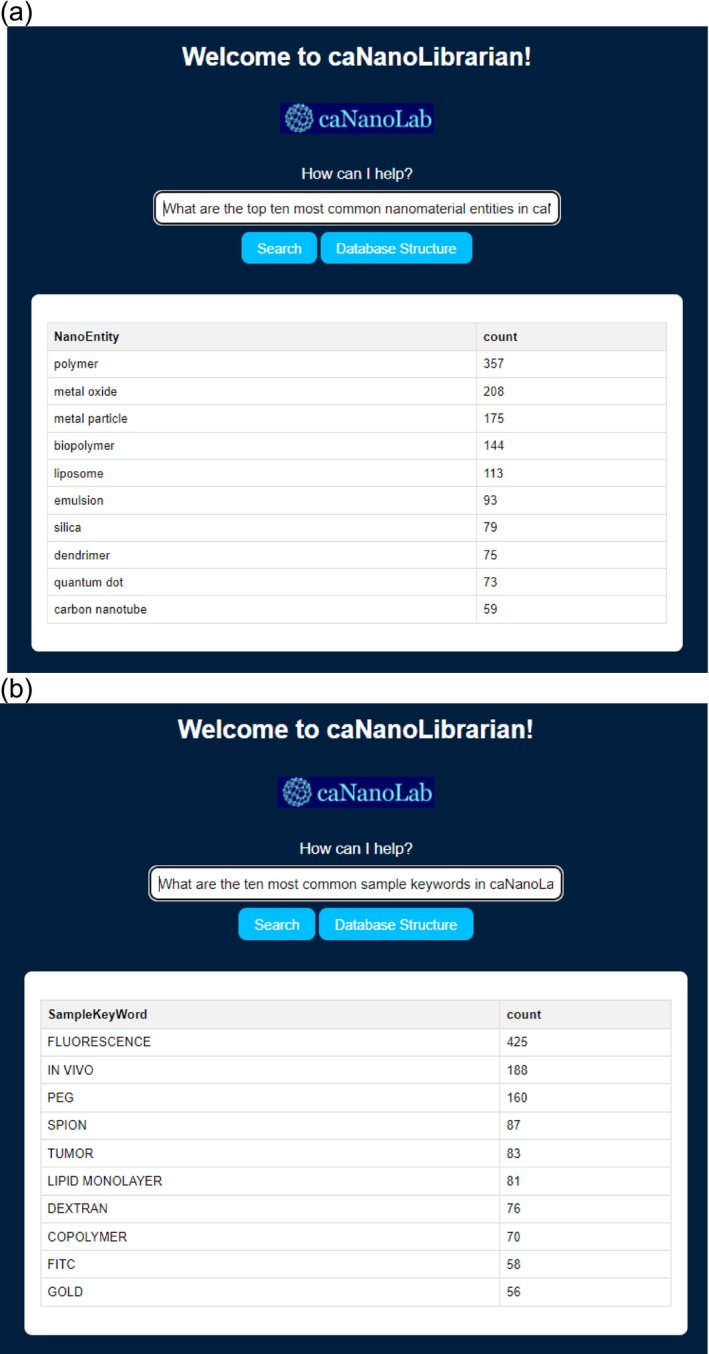
The search and analysis results are based on the user's input questions. Example of analysis results obtained using caNanoLibrarian. The top ten most common nanomaterial entities in caNanoLab (a) and the ten most common sample keywords in caNanoLab (b).

Further in this model, users can query the caNanoLab database using simple natural language, offering an alternative to the traditional search bar (Figure 7). In the traditional search bar approach, users would have to download the nanomaterial data for all samples and manually create a pivot table to identify the top nanomaterials. However, with the use of LLM, a simple question generates a visualized table, providing users with immediate results. Another example pertains to determining the most frequently used keywords in caNanoLab. Currently, the search results do not contain keyword information, making it challenging to obtain this data. However, LLM can address this query simply by asking a question, allowing users to access the desired information effortlessly.

One limitation of this model is that the LLM used is a generic language model, rather than one specifically trained for the biomedical field. Even if notes are added to the model, these notes may not cover all aspects of the field. Consequently, its comprehension abilities in biomedical contexts may be affected, potentially resulting in limitations in accurately understanding and interpreting complex biomedical‐related questions. However, as the development of LLM progresses, and more specialized training data becomes available, its capacity to comprehend the biomedical context is expected to improve (Chen et al. 2023). Other potential limitations include the model's dependence on prompt quality, which means that vague or poorly constructed questions may yield suboptimal results. Additionally, the model does not inherently verify the accuracy of its outputs, which may lead to inaccurate or outdated information if not cross‐checked. Another important consideration is the user interface and interaction: while the underlying technology is powerful, usability and user understanding of how to frame effective queries still pose practical barriers. However, with current advancements in engineering around LLMs, such as agentic workflow, incorporating tools for content verification and quality checks for results can help mitigate the inaccuracy problem. This is accompanied by increasing operating costs and a slowing of response speed, which could be offset through engineering choices. It is also important to note that there is no difference in using web‐based tools across platforms such as Mac and PC; the model performance and outputs are consistent. Furthermore, the accuracy of the search results is influenced by two key factors: the quality of the underlying data and the capability of the database infrastructure. Ensuring high‐quality data inputs and a robust database infrastructure is crucial in enhancing the accuracy and reliability of the search results provided by the model.

This deployment of the search bar powered by LLM brings unprecedented benefits, revolutionizing the way researchers interact with and leverage the extensive resources within the caNanoLab database. To get a firsthand experience of how this operates, we have prepared an illustrative example in Section 5.4. The introduction of an LLM‐powered search bar in the caNanoLab database aims to enhance how researchers find and utilize its resources, as illustrated in Section 5.4.

### 5.4 Experience caNanoWikiPDA and caNanoLibrarian–Your AI‐Powered Assistants

While we anticipate enhancements, actual benefits will be determined by performance analysis based on users' feedback, which can be collected via caNanoWikiPDA and caNanoLibrarian as follows: the user can visit caNanoWikiPDA at https://huggingface.co/spaces/ruiheCat/caNanoWiki and explore.

caNanoLibrarian at https://huggingface.co/spaces/ruiheCat/caNanoLibrarian.

Both of these applications are available for free through the GPT store at https://chat.openai.com/g/g‐aqgbJmhra‐cananolibrarian and https://chat.openai.com/g/g‐Jzez0Zf7c‐cananowiki.

Future work in this area may include fully implementing the auto‐curation pipeline and expanding its capabilities to encompass a broader range of nanotechnology studies, beyond oncology. This progress will involve leveraging LLMs to automate data extraction, enhance standardization, and maintain continuous updates through a four‐phase pipeline: automated curation, seamless integration, comprehensive coverage, and real‐time updates, ensuring that caNanoLab remains both comprehensive and current.

This advancement will be driven by the integration of three interconnected tools: the caNanoLab Wiki, caNanoLibrarian, and the auto‐curation pipeline. The Wiki offers user‐friendly guidance on navigating the system, understanding key terminology, and adhering to data submission standards, thereby making the platform more accessible and consistent. The auto‐curation pipeline ensures that caNanoLab can rapidly incorporate new findings by automating the ingestion and standardization of data from newly published studies, dramatically improving both the volume and freshness of curated content.

Building on this enriched data foundation, caNanoLibrarian may not only enable intelligent, context‐aware data retrieval but also support data interpretation and informed decision‐making. With the help of caNanoLibrarian, the application of caNanoLab can potentially be extended to include cross‐disciplinary searches through additional tools, such as internet searching or access to relevant databases, including those that link analyses of cancer nanomedicine data to nanotechnology applications for other diseases. By surfacing relevant datasets, identifying trends, and suggesting research directions, caNanoLibrarian enables researchers to design more effective experiments, uncover hidden connections, and pursue translational applications.

We envision that this integrated ecosystem, when grounded in a well‐defined nanotechnology dataset with comprehensive field coverage and rigorously validated by both AI and human experts, will lay the foundation for a high‐quality, interoperable data environment. In the future, it is expected to accelerate discovery, foster collaboration, and enable predictive analysis and interdisciplinary insight in nanomedicine, thereby contributing to and streamlining non‐clinical development of nanomedicines.

## 6 Conclusion

The integration of LLMs in cancer nanotechnology holds tremendous promise for addressing the challenges in data analysis, sharing, and management. Through advanced natural language processing techniques, LLMs have demonstrated their potential to enhance scientific data curation by extracting relevant information from unstructured text data and transforming it into structured formats. This automated curation process streamlines data management and facilitates the integration of valuable insights into databases like caNanoLab, ultimately contributing to the advancement of cancer nanotechnology research and its applications in diagnosis and treatment. These models offer researchers new tools for efficiently analyzing complex data, promoting collaboration, and accelerating scientific discoveries. By leveraging large language models, researchers can extract meaningful information from a vast amount of biomedical literature, thereby enabling a deeper understanding of the potential of nanotechnologies in cancer research. The ability to quickly access and analyze relevant scientific data using LLMs empowers researchers to make informed decisions, identify trends, and generate novel hypotheses, ultimately leading to innovative approaches and advancements in cancer diagnostics, therapeutics, and personalized medicine. Furthermore, LLM‐powered chatbots and natural language search functionalities can enhance user experience by providing prompt responses, personalized interactions, and intuitive access to information within platforms like caNanoLab. Researchers can leverage chatbots to efficiently obtain specific information and guidance, navigate complex databases, and receive real‐time support, ultimately streamlining their research processes. Natural language search capabilities enable users to query the caNanoLab database using everyday language, simplifying the search process and fostering a more user‐friendly experience. These features, combined with the potential for visualized data exploration and analytics, can empower researchers to uncover hidden patterns, discover new correlations, and gain deeper insights into cancer nanotechnology data.

The current implementation of LLMs through caNanoWikiPDA and caNanoLibrarian is a tangible step forward towards leveraging these advancements for the benefit of researchers and users in the field of cancer nanotechnology. While still in their early stages, these tools aim to provide accessible entry points to extensive datasets, simplify data curation processes, and offer real‐time support. Although user adoption is currently limited, we aim to continue developing and integrating these features to pave the way for enhanced collaboration and data accessibility in the future.

Looking ahead, there are exciting future directions for LLMs in cancer nanotechnology. Continued research and development will focus on refining LLM algorithms and expanding their capabilities to address domain‐specific challenges. Advancements in data analysis techniques, including the integration of domain knowledge and specialized training data, will improve the accuracy and comprehension of LLMs in the biomedical context. Additionally, efforts to enhance data‐sharing capabilities and interoperability will promote collaboration and data integration across research communities, fostering a more holistic and comprehensive understanding of cancer nanotechnologies. However, it is important to acknowledge the challenges and limitations that accompany the implementation of LLMs in cancer nanotechnology. These include the need for high‐quality training data, addressing data and response biases, ensuring data privacy and security, and addressing ethical considerations in the use of AI technologies (COTA Team 2023; Malek et al. 2022; Quotium). Ongoing research and collaboration between scientists, engineers, and ethicists will be essential to overcome these challenges and ensure the responsible and effective integration of LLMs in cancer nanotechnology research.

In conclusion, the integration of LLMs in cancer nanotechnology represents a potentially transformative approach to data analysis, sharing, and management. The potential for LLMs to enhance user experience and facilitate data exploration is substantial, paving the way for advancements in cancer research. By capitalizing on the capabilities of LLMs and addressing the associated challenges, researchers can unlock new insights and accelerate progress in the development of innovative nanotechnologies for cancer diagnosis and treatment. Ultimately, the future vision for LLMs in cancer nanotechnology is to empower researchers to leverage the capabilities of advanced AI technologies, revolutionizing cancer research and enhancing patient outcomes.

## Author Contributions

**Weina Ke:** conceptualization (equal), data curation (equal), formal analysis (equal), investigation (equal), methodology (equal), writing – original draft (equal). **Rui He:** conceptualization (equal), data curation (equal), formal analysis (equal), investigation (equal), methodology (equal), software (equal), writing – original draft (equal), writing – review and editing (equal). **Mark A. Jensen:** data curation (equal), funding acquisition (equal), resources (equal), supervision (equal), writing – review and editing (equal). **Marina A. Dobrovolskaia:** funding acquisition (equal), project administration (equal), resources (equal), supervision (equal), visualization (equal), writing – original draft (equal), writing – review and editing (equal).

## Conflicts of Interest

The authors declare no conflicts of interest.

## Data Availability

Data sharing is not applicable to this article as no new data were created or analyzed in this study.
